# CBCT data relevant in treatment planning for immediate maxillary molar implant placement

**DOI:** 10.34172/japid.2021.016

**Published:** 2021-11-21

**Authors:** Douglas Deporter, Maziar Ebrahimi Dastgurdi, Azadeh Rahmati, Eshetu G. Atenafu, Mohammad Ketabi

**Affiliations:** ^1^Department of Periodontology, Faculty of Dentistry, University of Toronto, Toronto, Canada; ^2^Faculty of Dentistry, University of Toronto, Toronto, Canada; ^3^Oral and Maxillofacial Radiology Center, Lahijan, Iran; ^4^Department of Biostatistics, University Health Network, Toronto, Canada; ^5^Department of Periodontology and Implant Dentistry, Islamic Azad University, Isfahan Branch, Iran

**Keywords:** Cone-beam computed tomography, immediate implant, inter-radicular septum, maxillary molar

## Abstract

**Background:**

This study used CBCT images to evaluate the suitability of maxillary first and second molar sites to receive immediate implants. Buccopalatal and mesiodistal widths of maxillary molar inter-radicular septum were evaluated at three different levels (crestal, middle, and apical), in addition to assessments of the root apex and furcation proximities to the sinus floor and comparisons of these measurements between the first and second upper molar sites before extraction.

**Methods:**

A total of 427 dental sites from 223 patients were used to measure the buccopalatal and mesiodistal widths of inter-septal/furcal (IRS) bone of maxillary first and second molars and vertical distances from the furcation and from all the root apices to the sinus floor (SF).

**Results:**

Mean coronal-most buccopalatal/mesiodistal IRS widths were 7.33/6.52 mm for the first and 6.86/5.85 mm for the second molars (P=0.008). Corresponding mean FSD (furcation-sinus floor) values were 9.69 mm (range: 2.02-24.68 mm) and 8.84 mm (range: 1.48-25.09 mm). Mean distances from all the root apices to SF were <3 mm. The palatal roots of the first molars had higher sinus intrusion rates (28.85%) than their buccal counterparts, while for the second molars, the mesiobuccal roots showed the highest sinus intrusion (37.65%).

**Conclusion:**

In the current patient sample, 61.7% of the first and 34% of the second molars had a sufficiently broad IRS to encase a 5-mm-diameter IMI (immediate molar implant) completely. The mean FSD of 9 mm for both molars indicated that some sinus floor elevation would likely be needed unless short implants were used.

## Introduction

Cone-beam computed tomographic (CBCT) scans are now universally considered the gold standard for implant site assessment and treatment planning.^1^ These three-dimensional cross-sectional images help the surgeon optimize implant positioning and avoid complications.^2^ The placement of dental implants at the time of tooth extraction (immediate implantation) was introduced in 1989 by Lazzara^3^ and is now widely used. The advantages of this approach include a reduction in the number of surgical interventions and overall treatment times.

When screening for immediate molar implant (IMI) treatment in the maxilla, some important pre-extraction radiographic parameters that need consideration include the dimensions of inter-radicular septal bone (IRS), the distance (bone height) between the molar furcation and sinus floor (SF), the distance from SF to each root apex, and any root intrusion into the sinus.

Within a few months following tooth extraction, substantial losses in vertical and buccopalatal alveolar ridge widths are expected to occur at maxillary molar sites.^4^ Furthermore, when more than one molar is lost in a maxillary sextant, sub-antral bone height losses of another 2 to 5.27 mm can occur.^5^ Any associated post-extraction sinus pneumatization might further limit the bone available for future placement of implants to replace lost molars with often greater pneumatization at maxillary second versus first molar sites and where two or more adjacent posterior teeth have been removed.^6^ Once all these changes have occurred, traditional delayed dental implant placement often becomes challenging, invasive, and costly. As a result, keen interest has developed in employing immediate maxillary molar implant treatment if certain prerequisites can be met. Systematic literature reviews with meta-analyses have suggested that IMIs have similar outcomes for implants placed in healed extraction sites.^7,8^ To date, however, the use of maxillary immediate molar implants has not been widely prescribed because of technical difficulties.^9^

The key to success with maxillary IMI treatment is careful site selection. A pre-treatment (i.e., before extraction) CBCT radiographic assessment is essential to estimate the remaining dimensions of the inter-radicular septal bone (IRS) associated with the molar furcation. Smith and Tarnow categorized molar socket IRSs into three types depending on their usefulness in stabilizing an implant of sufficient diameter to support a molar crown.^10^ Type A extraction sites have adequate IRS volume to completely contain the coronal perimeter of an appropriately sized implant (i.e., ≥5 mm in diameter) and are the most favorable sites for maxillary IMIs. In contrast, Type B sites are those that can stabilize the implant but not contain it completely. Finally, the IRS at Type C sites will have an insufficient volume to allow osteotomy localization within it. As a result, achieving stability of an IMI intended for a Type C socket will require either an even wider diameter implant to make some contact with the socket walls or placement of a longer implant into the palatal root socket if adequate bone remains apically to stabilize it.^11^ The mean available heights from furcation to sinus floor have been reported to be 7.43 mm for maxillary first and 7.07 mm for maxillary second molars. In contrast, an inter-radicular septum (IRS) is present in 74% of maxillary first and 44% of maxillary second molars.^12^

Smith & Tarnow classification can be roughly utilized as a useful clinical guide for placing appropriate sizes of dental implants into the IRS. However, there appears to be no published numerical data to help clinicians define IRS dimensions before extraction for the three socket types established by Smith and Tarnow and how these dimensions might vary between first molar (MFM) and second maxillary molar (MSM) sites. Previous studies using coronal and sagittal CBCT images have reported average distances from root apices to the sinus floor,^13-16^ and between molar furcation and sinus floor.^17^ However, none have addressed any dimensional differences in these parameters between first and second molars. Accordingly, the present study used CBCT data to assess the buccopalatal and mesiodistal widths of maxillary molar IRS at three different levels (crestal, middle, and apical) as well as assessments of both root apex and furcation proximities to the sinus floor (SF) and comparisons of these measurements between first and second molar sites before extraction.

## Methods

The CBCT images used for this retrospective study were obtained from the files of 223 patients seeking dental treatment in two different maxillofacial radiology centers in Lahijan, Iran, and Toronto, Canada, between 2016 and 2019. All the patients signed a consent form permitting that their CBCT images could be used for the measurements necessary for this research project. In total, 427 dental sites were eligible based on the fact that maxillary permanent first and/or second molars were present. Criteria for inclusion in the study were as follows:

1. Age >18 years
2. The presence of at least two occluding maxillary posterior teeth (premolar and/or molar) at least one of which was a fully erupted molar with fully formed root apices
3. No radiographic/clinical evidence of sinus pathology
4. No radiographic evidence of periodontal disease (bone loss), infection, severe root resorption, or periapical pathology
5. No history of previous surgical interventions of the selected teeth
6. Absence of metal restorations in the molar teeth that could create artifacts affecting the desired CBCT measurements
7. No current drug or alcohol abuse issues, history of chemotherapy, or relevant radiotherapy
8. No history of medications affecting the skeletal system

All the scans were taken using the same CBCT machine (Sirona Dental Systems, Bensheim, Germany) set at 98 kVp and 12 mA and assessed using Galileos viewer 1.9 software program (Sirona Dental Systems, Bensheim, Germany). To assess examiner reliability, all the images were measured twice within a 4-week interval. All the images were examined using a strict protocol after first rotating them such that their long axes were perpendicular to the occlusal plane. The axial CBCT view of the central fossa of each molar assisted in selecting the desired coronal and sagittal slices.

**Figure 1:**
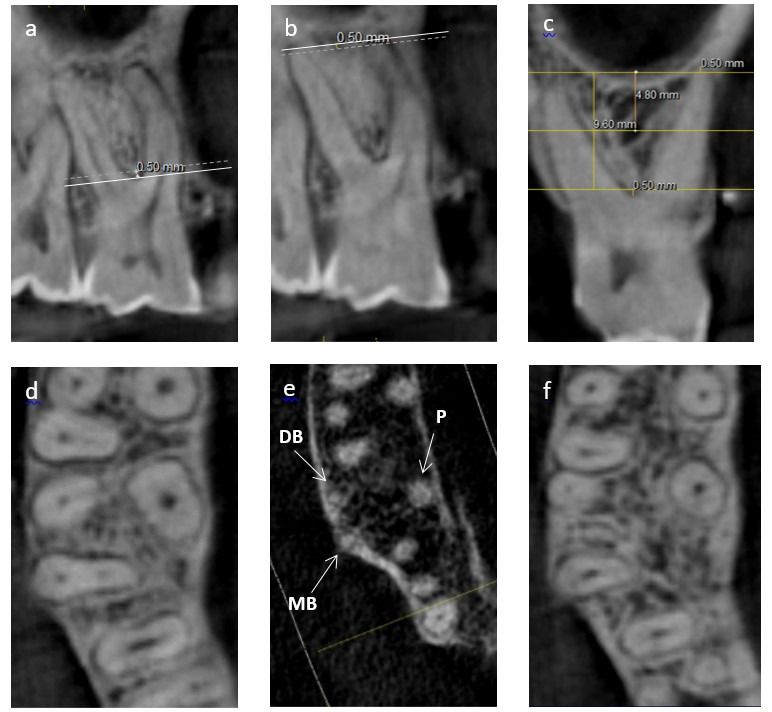
(a) A crestal measurement was recorded 0.5 mm apical to the molar furcation. (b) An apical measurement was recorded 0.5 mm coronal to the line connecting the apices of the two shortest roots. (c) A middle measurement was recorded midway between the crestal and apical levels; (d), (e), and (f) show the axial views of the same points corresponding to a, b, and c, respectively (MB: mesiobuccal, DB: distobuccal, P: palatal).

The following measurements were obtained from the CBCT images of each tooth:

Using axial images, IRS widths were recorded both mesiodistally and buccopalatally at three levels, i.e., crestal widths at 0.5 mm apical to the furcation, apical widths recorded at 0.5 mm coronal to a line connecting the apices of the two shortest roots, and middle level measurements taken at the mid-point between the other two measurement levels (Figure 1).

**Figure 2:**
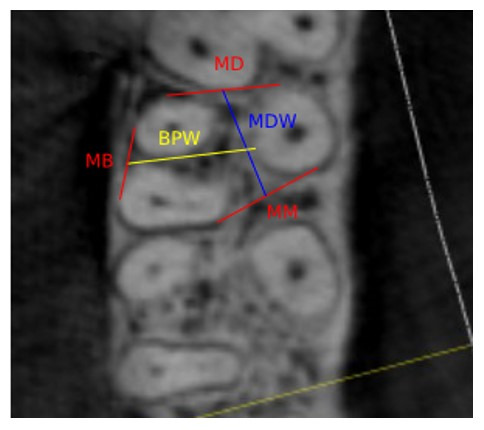
Buccopalatal width (BPW) and mesiodistal width (MDW) measurements were recorded at each of the crestal, middle, and apical levels of each molar IRS.

To allow impartial measurements at each of the three levels of data collection, reference lines were placed, as shown in Figure 2. To do this, the most mesial points of the mesiobuccal and palatal roots of the tooth were determined and connected by a straight line, and its halfway point was denoted as point mid-mesial (MM). Similarly, a straight line connecting the most distal points of the mesiodistal and palatal roots was used to establish a point called mid-distal (MD), and another connecting the most buccal aspects of the two buccal roots was used to establish the point designated mid-buccal (MB). After that, the distance between points MM and MD was recorded as the mesiodistal width of the IRS (MDW). Similarly, the buccopalatal IRS width (BPW) was recorded as the measurement from MB to the most buccal point of the palatal root.

**Figure 3:**
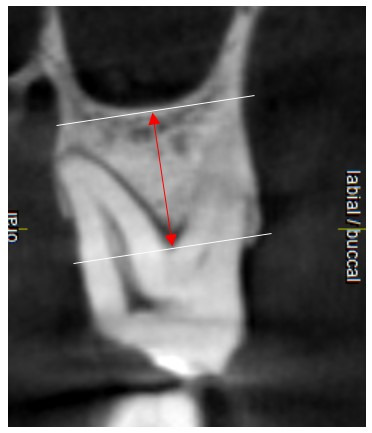
A measurement of the distance (FSD) from the furcation to the sinus floor (SF) was made in the middle-most coronal CBCT slice.

The distance (FSD) from the sinus floor (SF) to the molar furcation in the mid-most CBCT slice was recorded as described by Choi et al (Figure 3).^17^

**Figure 4:**
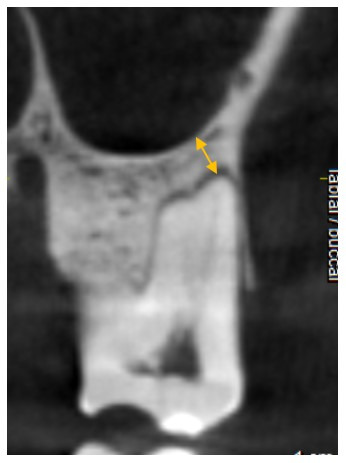
This figure shows an example of the measurement recorded for the distance of the mesiobuccal root apex to SF. In all the images, the closest point to SF was selected.

The shortest distance to SF from each root apex was recorded in the coronal slice (Figure 4).

The abbreviations for the parameters measured are as follows:

FSD = furcation to sinus floor distance

CBPD = crestal buccopalatal septum diameter

MBPD = middle buccopalatal septum diameter

ABPD = apical buccopalatal septum diameter

CMDD = crestal mesiodistal septum diameter

MMDD = middle mesiodistal septum diameter

AMDD = apical mesiodistal septum diameter

MBRS = mesiobuccal root apex-to-sinus distance

DBRS = distobuccal root apex-to-sinus distance

PRS = palatal root apex-to-sinus distance

Continuous variables of the first and second molars were compared with Student’s t-test. Pearson’s correlation analysis was used to assess any collinearity in the listed measures for each molar. Examiner reliability was assessed by the intraclass correlation coefficient. Linear mixed model analysis was employed to assess any differences in measures of MBRS, DBRS, and PRS to accommodate for the collinearity of these measurements from the same molar. Examiner reliability was evaluated at a statistical significance of P<0.05. Version 9.4 of Windows’ SAS was employed for all the analyses.

## Results

The intraclass correlation coefficient was 0.91. Table 1 summarizes continuous variables for the different CBCT measurements taken at maxillary first and second molars, as means, standard deviations, medians and/or ranges.

**Table 1:** The CBCT measurements taken at maxillary first and second molars are summarized as means, standard deviations, medians, and/or ranges

	**First Maxillary Molars**				**Second Maxillary Molars**			
	**N**	**Mean (+SD)**	**Min**	**Max**	**N**	**Mean (+SD)**	**Min**	**Max**
**FSD**	201	9.69 (4.29)	2.02	24.68	201	8.84 (3.54)	1.48	25.09
**CBPD**	203	7.33 (1.22)^a^	1.54	10.83	200	6.86 (1.63)^b^	1.3	12.78
**MBPD**	203	8.39 (1.24)^a^	2.61	11.03	201	7.69 (1.78)^b^	2.58	13.23
**ABPD**	203	9.24 (1.62)^a^	3.20	12.61	201	7.54 (2.19)^b^	1.66	11.84
**CMDD**	203	6.52 (1.2)^a^	2.18	8.89	200	5.85 (1.36)^b^	1.79	8.94
**MMDD**	203	6.17 (1.11)^a^	2.65	8.63	201	5.34 (1.38)^b^	1.95	8.38
**AMDD**	203	5.22 (1.4)^a^	2.18	8.41	201	3.97 (1.55)^b^	1.34	7.79
**MBRS**	203	3.06 (4.44)^aA^	-5.59	17.3	199	1.34 (3.52)^bA^	-5.56	16.02
**DBRS**	200	2.78 (4.14)	-4.20	16.11	201	2.68 (3.43)^B^	-3.90	14.41
**PRS**	201	2.54 (4.58)^B^	-5.68	17.42	201	2.96 (3.57)^B^	-4.57	14.58

Different capital letters indicate significant differences in vertical lines, and different lowercase letters indicate significant differences in the horizontal lines. furcation to sinus floor distance (FSD), crestal buccopalatal septum diameter (CBPD), middle buccopalatal septum diameter (MBPD), apical buccopalatal septum diameter (ABPD), crestal mesiodistal septum diameter (CMDD), middle mesiodistal septum diameter (MMDD), apical mesiodistal septum diameter (AMDD), mesiobuccal root apex-to-sinus distance (MBRS), distobuccal root apex-to-sinus distance (DBRS), palatal root apex-to-sinus distance (PRS)

### Estimation of the inter-radicular septum (IRS) diameters

The mean crestal (CBPD), middle (MBPD), and apical (ABPD) buccopalatal IRS widths for maxillary first molars were 7.33, 8.39, and 9.24 mm, respectively, compared to those for second maxillary molars, i.e., 6.86, 7.69, and 7.54 mm. These mean values were significantly different (P=0.008, P=0.003, and P<0.0001, respectively) between the first and second molar sites. Regarding mesiodistal widths of IRS, the mean crestal (CMDD), middle (MMDD), and apical (AMDD) values for maxillary first molars were 6.52, 6.17, and 5.22 mm, respectively, compared to the corresponding values for second molars (5.85, 5.34, and 3.97 mm); and, once again all these parameters were significantly different between the two molar sites (P=0.014, P<0.0001, and P<0.0001, respectively). It was of interest for us to estimate the percentage of molar IRS values that could be classified as Types A, B, and C. In their original classification, Smith and Tarnow^10^ did not provide actual measurements for each IRS type. Rather, they based the differences on whether or not the crestal aspect of a 5-mm-diameter implant could be inserted to be completely encased in bone or not. Therefore, in the present investigation, we arbitrarily assigned dimensions for each IRS type as follows: Type A to have >6 mm of bone both M-D and B-P; Type B to have between 3 mm and 6 mm of M-D or B-P and Type C to have <3 mm of IRS bone M-D or B-P. This allowed us to estimate the percentages shown in Table 2.

**Table 2:** Percentage of Types A, B, and C forfirst and second molars

	**First Maxillary Molars**	**Second Maxillary Molars**
**Type**	**%**	**%**
**Type A**	61.7	34.03
**Type B**	33.45	59.41
**Type C**	4.85	6.56

### Furcation to sinus floor distances (FSD)

The mean FSD distance for maxillary first molars was 9.69 mm, with a range of 2.02-24.68 mm. The corresponding mean value for second molars was 8.84 mm (range: 1.48-25.09 mm). These distances were not significantly different (P=0.539). These FSD distances were categorized into <5, 5-9, and >9 mm (Table 3). Based on these measurements, and assuming that 5 mm of FSD is sufficient to place an implant without the need for direct sinus floor elevation, fewer second molar sites would require this more invasive treatment.

**Table 3:** Categorization of FSD measurements for both molar locations

	**First Maxillary Molar**	**Second Maxillary Molar**
**FSD**	**%**	**%**
**<5mm**	17.58	9.16
**5-9mm**	21.23	49.17
**>9mm**	61.19	41.67

Furcation to sinus distance (FSD)

### The distance from the root apex to the sinus floor

Mean distances from the mesiobuccal (MBRS), distobuccal (DBRS), and palatal (PRS) root apex to SF were 3.06, 2.78, and 2.54 mm for first molars and 1.34, 2.78, and 2.54 mm for second molars. Only the MBRS distances were significantly different (P=0.035) between the two molar sites.

### Root intrusion

Regarding root intrusion into the sinus, 38.94% of first and 45.45% of second molars had one or more roots showing intrusion into the sinus (Table 4). The palatal roots of maxillary first molars showed higher intrusion rates (28.85%) than the two buccal roots, while with second molars, the mesiobuccal roots were more likely to intrude into the sinus (37.65%) (Table 5).

**Table 4:** Descriptive statistics of root intrusion for first and second maxillary molars mesiobuccal root apex-to-sinus (MBR), distobuccal root apex-to-sinus (DBR), palatal root apex-to-sinus (PR) distances

		**With Intrusion**				**Without Intrusion**			
**Tooth**		**Percentage**	**Min**	**Max**	**Mean (±SD)**	**Percentage**	**Min**	**Max**	**Mean (±SD)**
**First Maxillary Molars**	**MBR**	20.18	–5.59	–0.31	–1.81(1.37)	79.82	0.21	17.3	5.15(3.93)
	**DBR**	24.51	4.20	–0.11	–1.87(1.48)	75.49	0.23	16.11	4.89 (3.85)
	**PR**	28.85	–5.68	–0.14	–1.61(1.4)	71.15	0.18	17.42	5.37(3.81)
**Second Maxillary Molars**	**MBR**	37.65	–5.56	–0.19	–1.61(1.09)	63.35	0.11	16.02	3.13(3.26)
	**DBR**	20.48	–3.90	–0.22	–1.84(1.21)	79.52	0.05	14.41	3.85(2.91)
	**PR**	22.89	–4.57	–0.14	–1.37(1.17)	77.11	0.04	14.58	4.02(3.13)

**Table 5:** Percentages of first and second maxillary molars with no or one, two, and three root apex intrusions

	**First Maxillary Molar**		**Second Maxillary Molar**	
**Number of** **Intruded roots**	**Percentage**	**Cumulative** **Percentage**	**Percentage**	**Cumulative** **Percentage**
**1**	18.58	18.58	27.27	27.27
**2**	9.73	28.32	11.36	38.64
**3**	10.62	38.94	6.82	45.45
**No intrusion**	61.06	100.00	54.55	100.00

## Discussion

There are some key clinical factors affecting outcomes following immediate molar implant (IMI) placement.^18^ One of them is socket anatomy, which will dictate whether an IMI can be properly stabilized.^19,20^ Optimal, prosthetically driven IMI positioning is often best achieved if the implant osteotomy is prepared into the inter-septal/furcal bone (IRS). The results of the present study determined the mean coronal-most, buccopalatal, and mesiodistal IRS widths at 7.33 and 6.52 mm for maxillary first molars and 6.86 and 5.85 mm for second molars, respectively. In addition, buccopalatal widths of IRS increased towards the apical level while mesiodistal IRS widths decreased apically (see Table 1).

In their classification of maxillary molar socket anatomies, Smith and Tarnow^10^ designated Type A sockets to be those into which an implant of at least 5-mm diameter can be placed into the remaining IRS such that its entire coronal portion, when seated, is fully surrounded by bone. Combining the assumption of Smith and Tarnow with our new data, we suggest that Type A sockets are those with IRS widths >6 mm.

Assuming that >6 mm of IRS width buccopalatally and/or mesiodistally would be required to house a 5-mm-diameter implant, we estimated that in our sample of patients, 61.7% of maxillary first molars and 34.34% of second molars were qualified as Type A. For Type B sockets, i.e., those classified by Smith and Tarnow as having sufficient IRS bone to stabilize an IMI but insufficient to encase its coronal portion fully, we assigned the measurement range between 3 and 6 mm. Based on these values, it was estimated that 33.45% of first and 59.41% of second molar sites were Type B in our sample. Only 4.85% of first and 6.56% of second molar sockets in our sample were Type C, which we considered to be those <3 mm in width.

In their latest report, Smith et al^20^ reported retrospective outcomes for 300 IMIs placed in either jaw (specific numbers per jaw not given) from 2006 to 2017, including a technique development phase. Flap-less surgery during extraction and no peri-implant gap grafting or barrier membranes were used. Only twenty Type A sockets were identified, suggesting that more mandibular than maxillary sites and/or more second (see there Figures 7 to 13) than first molar sites were treated. The majority were Type B (185) or Type C (95) sockets, and while failure was <3%, all failures were in Type B sockets. The authors did not speculate on why Type B sockets were the most challenging. Implants from five different manufacturers were used with lengths and diameters ranging from 8.5 to 13 mm and 4 to 9 mm, respectively. Only eighteen of the 300 implants were 4 mm in diameter, with 106 being ≥6 mm in diameter and the remainder between 4.6 and 5.8 mm in diameter. They reported that with Type C and some Type B sockets, insufficient IRS meant that a crucial factor for success was the need for intact, thick outer bony socket walls and use of implants of wide enough diameter to contact these walls; interestingly, only one of the 106 implants measuring ≥6 mm in diameter failed.

The mean FSD height, i.e., bone height between the molar furcation and sinus floor, of IRS in the present sample was 9.69 mm for first and 8.84 mm for second molar sockets. However, others have reported that provided that FSD is ≥5 mm at a healed molar extraction socket that an implant can be placed without the need for direct sinus floor elevation techniques.^21^ If one assumes that an FSD of ≥5 mm is also suitable for placing an IMI, then 82.42% of the first molar and 90.84% of the second molar sockets in the present sample could be considered favorable.

If the clinician concludes that there is insufficient IRS bone volume to secure an IMI (Types B or C), one option might be to place the implant into the tooth’s palatal root socket using the bone available apically to gain primary stability.^22^ However, mean distances from the palatal root apices to the sinus floor in the present sample were <3 mm. In addition, palatal root intrusion into the sinus needs consideration since 28.85% of first molar palatal root apices showed such intrusion adding additional difficulty and risk. Less palatal root intrusion into the sinus (22.89%) was found at second molar sites, suggesting that implant placement into the palatal root here might be an option.

## Conclusions

Valuable pre-extraction information can be obtained from CBCT scans of maxillary molars being considered for immediate replacement with dental implants (IMIs). If sufficient inter-septal/furcal bone (IRS) remains, the ideal osteotomy location for an IMI is considered to be in the IRS. In the sample of patients considered in the present study, 61.7% of maxillary first molars, but only 34% of second molars, would have been potential candidates for this treatment approach. In addition, the mean bone heights from the furcation to the sinus floor (FSD) in the present sample for both molar sites was approaching 9 mm, indicating that unless a short implant^23^ was to be used, some sinus floor elevation, possibly using trans-crestal techniques, would be necessary at most sites.

## Authors’ contributions

Conceptualization, MK and DD; methodology, MK and DD; investigation, MED; resources, AR; writing the original draft, MK and DD; formal analysis, EA; writing the review and editing, MK and DD; supervision, DD.

## Avalibility of data

The data from the reported study are available upon request from the corresponding author.

## Ethics approval

The protocol of the present study was approved by the Ethics Committee of Guilan University of Medical Sciences under the code IR.GUMS.REC.1396.324. Written informed consent was obtained from all the patients for using their scans in this study.

## Competing interests

The authors declare that they have no competing interests regarding authorship and/or publications of this paper.
